# Assessing the Effects of Prior History of Vertebral Osteomyelitis on Peri-Operative Factors and Post-Operative Recovery in Adult Spinal Deformity Patients

**DOI:** 10.3390/jcm11216488

**Published:** 2022-11-01

**Authors:** Peter S. Tretiakov, Rachel Joujon-Roche, Tyler Williamson, Bailey Imbo, Claudia Bennett-Caso, Pooja Dave, Kimberly McFarland, Jamshaid Mir, Michael Dinizo, Andrew J. Schoenfeld, Peter G. Passias

**Affiliations:** 1 Departments of Orthopedic and Neurological Surgery, NYU Langone Health/New York Spine Institute, New York, NY 10016, USA; 2 Department of Orthopaedic Surgery, Brigham and Women’s Hospital, Harvard Medical School, Boston, MA 02115, USA

**Keywords:** adult spinal deformity, vertebral osteomyelitis, osteomyelitis

## Abstract

Background: Vertebral osteomyelitis (VOM) is a relatively rare infection of the vertebral body that requires aggressive antibiotics and may necessitate operative debridement, decompression, and/or fusion of affected segments. Post-treatment VOM can result in focal deformity, or can occur in conjunction with a global deformity. The influence of previous VOM on adult spinal deformity (ASD) surgery outcomes has not been examined previously. Purpose: To determine whether patients with a history of previous VOM treated for spinal deformities have different post-operative clinical trajectories or outcomes, including an increased risk of peri-operative complications and recurrence of infection. Study design/setting: Retrospective review. Patient sample: 836 ASD patients. Outcome measures: Complications; antibiotic course; HRQLs Methods: Patients (>18 y) with a history of resolved vertebral osteomyelitis (VOM) prior to primary deformity surgery, with complete data up to 2Y were included. A case–control analysis was performed, with cases of confirmed VOM (VOM+) matched to individuals without history of VOM (VOM−) in 1:1 fashion based on age, gender, and number of co-morbidities. Given the exploratory nature of this work, bivariate comparisons using chi-squared tests for categorical outcomes and t-test for continuous data were used. Results: 18 VOM+ patients were included (55.83 ± 10.42 years, 38% female, 29.48 ± 6.85 kg/m^2^). At baseline, VOM+ patients were significantly more likely to have a history of cancer (62.8 vs. 56.8, *p* = 0.011), and to be actively undergoing cancer treatment (*p* = 0.013) at the time of primary ASD surgery. HRQLs (*p* > 0.05) were similar between groups. In terms of baseline (BL) parameters, neither group demonstrated significantly different C-reactive protein (CRP), hemoglobin, or albumin (*p* > 0.05). Surgically, VOM+ patients did not have significantly higher mean levels fused (*p* = 0.002), mean blood loss (*p* < 0.001) or longer operative time (*p* = 0.003) compared to VOM− patients. Post-operatively, one VOM+ patient (5.6%) experienced recurrence of osteomyelitis in the thoracic spine after initially receiving treatment for lumbar VOM. VOM+ were found to have longer hospital length of stays (8.154 vs. 4.772 days, *p* = 0.003). At 2Y follow-up, there was no significant differences in terms of ODI, EQ5D/EQ5D-VAS, or NRS-Neck or NRS-Back (*p* > 0.05), rate of mechanical complications or surgical site infections (all *p* > 0.05). Conclusions: A history of previously treated VOM in adult spinal deformity patients appears to be associated with increased total hospital length of stay, blood loss, and operative time compared to case–control matched VOM− patients. Nonetheless, VOM+ pateints demonstrated iimprovement in terms of patient-reported outcomes without an increased risk of mechanical complications.

## 1. Introduction

Vertebral osteomyelitis (VOM), which includes the clinical entities of spinal osteomyelitis, spondylodiscitis, or pyogenic spondylitis, describes a complex inflammatory reaction within the vertebral column in the setting of microbial infection [1,2,3,4]. Even following effective treatment, VOM can result in vertebral body collapse, local deformity or worsening of previous global spinal deformity. As the prevalence of VOM is increasing for a variety of reasons within developed countries, this diagnosis among patients seeking treatment for adult spinal deformity is becoming more frequently encountered. Within the adult spinal deformity (ASD) patient population, the history of VOM prior to surgical correction has increased in incidence over time, with approximately 5–6 cases per 100,000 reported in recent studies [5,6].

Although there is little published data, many surgeons assume that a history of VOM can increase the complexity of deformity surgery can elevate the risk for adverse peri-operative events, including construct failures, development of proximal junctional kyphosis (PJK) and mechanical compromise. There is also concern that a history of VOM can elevate the risk of peri-operative mortality, as it has been recognized to be as high as 20% in certain clinical scenarios [6,7,8].

As spine surgeons attempt to limit peri-operative risk factors, the influence of a previously treated VOM on ASD surgery outcomes remains largely unknown. In this context, we conducted an exploratory analysis using a large, prospectively collected, dataset of patients with ASD who underwent surgical correction. This data source has been used previously to study both clinical and health policy aspects of ASD surgery [9,10,11]. We sought to determine whether patients treated for spinal deformities with a history of successfully treated VOM have a different post-operative trajectory, including an increased risk of complications and reduced health-related quality of life (HRQL). Based on clinical experience, we hypothesized that by 2 year follow-up, patients undergoing surgery for ASD with a history of VOM would be at increased risk for post-operative complications but may achieve similar HRQL improvements compared to VOM− patients.

## 2. Materials and Methods

We conducted an exploratory retrospective case series analysis of a prospectively collected, single-center database of adult spinal deformity (ASD) patients treated between 2014 and 2020. Institutional Review Board (IRB) approval was obtained prior to enrollment and all patients provided informed consent. Patients enrolled in the database were ≥18 years of age and underwent surgical correction for ASD. ASD was defined radiographically by the presence of at least one of the following parameters: coronal Cobb angle ≥20°, sagittal vertical axis ≥50 mm, pelvic tilt ≥25° and/or thoracic kyphosis >60°.

The inclusion criteria of the present study were operative ASD patients with complete radiographic and health related quality of life (HRQL) data preoperatively and at 2 years postoperatively. Patients with a cervical fusion during index surgery (upper instrumented vertebra [UIV] above T1) and patients who underwent a revision involving fusion of the cervical spine after index surgery were excluded. All patients assessed in this study presented with non-traumatic progressive scoliotic degeneration or de novo scoliosis. Patients with active spinal tumors were excluded, as were those with pure cervical deformity.

### 2.1. Data Collection and Radiographic Assessment

Standardized data collection forms assessed patient demographics, surgical parameters, and comorbidities at initial presentation. HRQL metrics were collected via patient surveys at baseline and at multiple follow-up time points. These included the Short Form-36 (SF-36), Oswestry Disability Index (ODI), and Scoliosis Research Society-22r (SRS-22r).

Anteroposterior and lateral spine radiographs were used to assess radiographic parameters at baseline and all follow-up intervals. All images were analyzed with SpineView^®^ (ENSAM, Laboratory of Biomechanics, Paris, France) [12,13]. Spinopelvic radiographic parameters assessed included pelvic tilt (PT: the angle between the vertical and the line through the sacral midpoint to the center of the two femoral heads), the mismatch between pelvic incidence and lumbar lordosis (PI-LL), and the sagittal vertical axis (SVA: C7 plumb line relative to the posteriosuperior corner of S1) [14].

### 2.2. Measures of Radiographic Alignment

Thoracolumbar deformity severity was assessed using the SRS-Schwab ASD classification system, which involves three established modifiers of PT, PI-LL, and SVA, each stratified into three tiers of severity: 0 [non-pathologic], + [moderate deformity], ++ [marked deformity] [15]. Age-adjusted alignment targets for sagittal correction were assessed using previously published formulas established by Lafage et al. [16]. Lastly, the Global Alignment and Proportion [GAP] score was calculated. This scoring system includes four parameters, namely relative pelvic version (measured minus ideal sacral slope) [0–3], relative lumbar lordosis (measured minus ideal lumbar lordosis) [0–3], lordosis distribution index (L4-S1 lordosis divided by L1-S1 lordosis multiplied by 100) [0–3], and relative spinopelvic alignment (measured minus ideal global tilt) [0–3], as well as an age factor [0–1]. The total score is calculated out of 13 and determines proportionality based on previously defined distribution (≤2 Proportional, 3–6 Moderately Disproportional, ≥7 Severely Disproportional [17].

### 2.3. Defining Cohorts

Patients (≥18 years) with a previous diagnosis of thoracolumbar vertebral osteomyelitis (VOM), along with documentation of VOM resolution at least one year prior to index surgery, with complete data up to 2Y were retrospectively analyzed. Active VOM was ruled out after patients had negative lab markers at 6 months and 1 year prior to surgery, and C-reactive protein (CRP), procalcitonin (PCT), and WBC levels remained within normal limits (WNL). Furthermore, comparative imaging including magnetic resonance imaging (MRI) was utilized, and no signal changes were noted in patients with resolved VOM. Patients were eligible for inclusion in this analysis regardless of operative or non-operative VOM treatment.

### 2.4. Statistical Analysis

A case–control analysis was performed, with cases of confirmed VOM (VOM+) matched to individuals without history of VOM (VOM−) in 1:1 fashion based on baseline age, gender, and number of co-morbidities (defined using the Charlson Comorbidity Index [CCI]) [18]. Given the exploratory nature of this work, and the size of the sample, we relied on bivariate comparisons using chi-squared tests for categorical outcomes and *t*-test for continuous data. Statistical significance was set for all analyses at *p* < 0.05.

## 3. Results

### 3.1. Cohort Overview

Among 836 total ASD patients in the registry, 18 (2.2%) patients were identified as having a history of treated VOM (55.83 ± 10.42 years, 38% female, 29.48 ± 6.85 kg/m^2^) and classified as VOM+. Mean Charlson Comorbidity Index (CCI) was 4.67 ± 2.66 (Table 1). At the time of operation, 56.2% of patients were classified as ASA grade II, 38.3% were considered grade III, and the remainder evenly split between grades I and IV. Of the observed cohort, 65.6% had a history of prior spine surgery of any kind. Mean preoperative radiographic parameters were as follows: SS 32.4°, PT 21.1°, PI-LL 10.5°, SVA 49.6 mm, TS-CL 19.8°, cSVA 28.4 mm, CBVA 7.2°, C2-T3 7.9°, and C2-C7 lordosis 7.7°. Of the observed cohort, 79.0% of patients had a previous history of lumbar VOM, while 21.0% had a history of thoracic VOM.

### 3.2. Radiographic and Surgical Characteristics

Radiographically, VOM+ patients presented with greater mean C7-S1 sagittal vertical axis (SVA) values (*p* < 0.001) at baseline. Furthermore, VOM+ patients presented with significantly higher mean pelvic tilt (*p* = 0.002), pelvic incidence (*p* = 0.023), as well as pelvic incidence minus lumbar lordosis (PI-LL) mismatch (*p* = 0.005) (Table 2).

In the overall ASD surgical cohort, mean levels fused were 7.88 ± 5.02, mean estimated blood loss (EBL) was 2406 mL, and mean operative time was 467 min. By surgical approach, 5.6% of patients underwent anterior-only, 81.8% underwent posterior-only and 9.1% combined approach, with no significant difference in approach between cohorts (all *p* > 0.05). Overall, 12 (66.7%) VOM+ patients underwent osteotomy as part of their index procedure, while 1 patient (5.6%) underwent laminectomy and 11 (61.1%) underwent limited decompression. Of the patients studied, 88.9% had their level of previous VOM included in the fusion construct.

### 3.3. Baseline Characteristics Comparison

At baseline, VOM+ patients were significantly more likely to have a history of cancer (62.8 vs. 56.8, *p* = 0.011), and to be actively undergoing cancer treatment (*p* = 0.013) at the time of index ASD surgery. However, VOM+ patients did not report worse HRQLs (all *p* > 0.05). In terms of baseline parameters, neither group demonstrated significantly different C-reactive protein (CRP), hemoglobin, or albumin levels (*p* > 0.05). Surgically, VOM+ patients had a significantly higher mean number of levels fused (*p* = 0.002) and operative time, and higher mean blood loss (*p* = 0.001) even when adjusting for levels fused and operative time (*p* = 0.021) compared to VOM− patients.

### 3.4. Peri-Operative Course

Compared to standard post-operative antibiotic prophylaxis, VOM+ patients were administered post-operative antibiotics for an average of 12.0 weeks. VOM+ patients were also found to have longer hospital length of stays (8.154 vs. 4.772 days, *p* < 0.001) compared to VOM− patients (Table 3).

### 3.5. Post-Operative Radiographic Analysis

Radiographic analysis of VOM− vs. VOM+ patients by 2-years post-operatively revealed no significant differences between groups in terms of C7-S1 SVA (*p* = 0.071) or PI-LL mismatch (*p* = 0.105). However, unadjusted comparison did reveal higher pelvic tilt (*p* = 0.029) and pelvic incidence (*p* = 0.045) in the VOM+ cohort (Table 4).

### 3.6. Post-Operative Complications Analysis

Post-operatively, one VOM+ patient (5.6%) experienced a recurrence of osteomyelitis in the thoracic spine after initially receiving treatment for lumbar VOM. One VOM+ patient was readmitted for deep wound infection, though case–control analysis did not report significant differences in subsequent superficial nor deep surgical site infections (all *p* > 0.05). By 2Y, VOM+ patients did not report significant differences in terms of ODI, EQ5D/EQ5D-VAS, NRS-Neck or NRS-Back (*p* > 0.05) (Table 5). There was no difference in mechanical complications or reoperation rates between groups (*p* > 0.05). No significant differences in ODI-Employment domain by 2Y between cohorts (*p* > 0.05), and in both groups the greatest proportion of patients reported that their back pain increased with regular tasks, or were prevented in performing only physically strenuous tasks.

## 4. Discussion

Vertebral osteomyelitis continues to present significant challenges to adult spinal deformity patients and surgeons, both in diagnosis and treatment. Previous studies have focused primarily on the treatment of acute VOM, and various treatment strategies have been described which include both non-operative and operative approaches. However, to the best of our knowledge, this study is the first of its kind to describe the effect of resolved VOM at least 1 year prior to adult spinal deformity surgery [19,20,21,22]. We sought to examine the effects of previous VOM on rates of recurrence, clinical and patient-reported outcomes, and complication rates in ASD surgery patients.

Previous larger-scale studies by McHenry et al. (2002) reported that of 253 patients with acute VOM and a median follow-up of 6.5 years, mortality was observed in 11 (4.3%) patients, and relapse occurred in 14% of patients [23]. In our study, we observed no mortality and only one patient who had recurrent VOM, despite relatively high Charlson Comorbidity Index (CCI) scores, possibly indicating that improvements in pre-operative medical optimization may play a greater role in reducing the risk of infection that previously thought. These findings also support the 2013 North American Spine Society’s (NASS) guidelines, which were utilized at this study site, which outlined best-practices for administering peri-operative antibiotic therapy to patients at risk of recurrent VOM infection [24].

In their review of temporal improvements in pathophysiological identification of VOM and advancements in treatment, Birt et al. also demonstrate that local administration of antibiotics may more accurately and effectively target the pathogens responsible for infection and reduce biofilm formation when compared to systemic high-dose antibiotic therapy [25]. However, both studies report high heterogeneity and limited high-quality data remain significant limitations. Predicting recurrent infection also remains an incredibly difficult practice, though Kim et al. does report that from their artificial neural-network analysis that serial C-reactive protein (CRP) measurement increases the sensitivity by approximately 56 to 61% compared to traditional logistic models, even when accounting for baseline patient risk factors for recurrence of VOM [26].

Despite the difficulties in preventing recurrent vertebral osteomyelitis, our findings demonstrate that patients with a history of VOM that is clinically treated prior to index surgery obtain excellent clinical outcomes and are comparable to patients without a history of previous VOM. These findings fall in line with previous analyses by Sleiman et al., who demonstrated that spinal infections may detract from clinical and patient-reported improvements within the immediate post-operative period, but beyond one-year post-operatively they do not have significantly different outcomes or revision rates compared to VOM− patient [27]. Temporal studies by Agarwal et al. further demonstrate that application of an infection prevention reimbursement bundle along with increased physician awareness, may decrease the risk of infection-related post-operative complications by 54%, while also increasing overall cost-effectiveness by an estimated 291,000 USD [28]. The combination of such modalities supports the findings of our study that for patients with a history of resolved vertebral osteomyelitis, surgical treatment remains a safe and effective method of treating adult spinal deformity.

## 5. Limitations

Though our findings suggest comparable outcomes in patients with a history of VOM compared to those without, we recognize several potential limitations. The retrospective single-center nature of the data source, increase the potential for selection, indication and cluster bias. The limited sample size is of primary concern and is underpowered with respect to detecting clinically important secondary affects and interactions. This creates some concern for residual confounding and necessitates future study with a larger sample size and the capacity for more robust adjustment. This may require multicenter collaboratives given the relatively low apparent incidence of ASD surgery in setting of VOM in our cohort. Second, infection control modalities may vary greatly by region, and multicenter analyses are needed to better understand global and regional trends in outcomes. Due to a paucity in current literature, studies assessing the type of VOM treatment provided prior to ASD surgery, and the impact on realignment, are also warranted. Furthermore, while we are able to report on patient-reported employment questionnaire responses, many patients included are on long-term disability, and our follow up data was limited. Therefore, we are unable to report exact time to return to work. Lastly, while we present viable data regarding maintenance of surgical outcomes with reasonable surgical follow up, longer-term surveillance up to 5– and 10–years following surgery is clearly necessary. We envision future work along these lines going forward. Despite these limitations, our data presents supporting evidence that, while possibly increasing the complexity of surgical correction, a history of VOM itself should not present a substantive barrier to ASD surgery for those patients who require it.

## 6. Conclusions

The present study demonstrates that total hospital length of stay, blood loss, operative time, and post-operative antibiotic duration appears to be elevated in adult spinal deformity patients with successfully treated vertebral osteomyelitis. Nonetheless, these individuals still demonstrate improvement in patient-reported outcomes without increased risk of mechanical complications or surgical site infection.

## Figures and Tables

**Table 1:** Baseline demographic comparisons of VOM+ and VOM− patients.

Mean Baseline Parameter	Cohort	Mean	Std. Deviation	Significance
Age (y)	VOM−	56.83	12.54	*p* = 0.785
	VOM+	55.91	11.55	
Body Mass Index (BMI) (kg/m^2^)	VOM−	31.05	6.72	*p* = 0.381
	VOM+	29.40	7.22	
Gender (% F)	VOM−	0.45	0.50	*p* = 0.440
	VOM+	0.47	0.52	
History of smoking (y/n)	VOM−	0.24	0.43	*p* = 0.690
	VOM+	0.17	0.41	
Preoperative prescription opioid use (%)	VOM−	0.60	3.12	*p* = 0.842
	VOM+	0.42	0.52	
Preoperative serum C-reactive Protein (CRP) (mg/L)	VOM−	5.77	7.37	*p* = 0.412
	VOM+	6.22	2.46	
Preoperative serum haemoglobin (g/dL)	VOM−	13.92	1.50	*p* = 0.090
	VOM+	12.92	2.53	
Preoperative serum albumin (g/dL)	VOM−	20.30	123.89	*p* = 0.297
	VOM+	1.92	6.93	

**Table 2:** Baseline radiographic comparison of C7-S1 plumbline (SVA), pelvic tilt (PT), pelvic incidence (PI), and pelvic incidence minus lumbar lordosis (PI-LL) of VOM+ and VOM− patients.

Baseline Radiographic Parameters	Cohort	Mean	Std. Deviation	Significance
Plumbline C7 to S1 (SVA, mm)	VOM−	62.76	70.42	*p* < 0.001
	VOM+	85.53	66.71	
Pelvic Tilt (PT, °)	VOM−	23.28	10.65	*p* = 0.002
	VOM+	26.16	10.28	
Pelvic Incidence (PI, °)	VOM−	55.21	13.07	*p* = 0.023
	VOM+	57.87	12.80	
Pelvic Incidence—Lumbar Lordosis (PI-LL, °)	VOM−	15.26	20.85	*p* = 0.005
	VOM+	20.02	19.69	

**Table 3:** Surgical and peri-operative comparisons of VOM+ and VOM− patients.

Mean Peri-Operative Factors and Complication Rates.	Cohort	Mean	Std. Deviation	Significance
Number of levels fused	VOM−	3.64	3.63	*p* < 0.001
	VOM+	8.79	5.07	
Operative time	VOM−	375.53	190.41	*p* = 0.011
	VOM+	498.00	127.77	
Estimated Blood Loss (mL)	VOM−	682.00	953.79	*p* = 0.002
	VOM+	1007.28	960.63	
Length of stay (days)	VOM−	4.77	4.28	*p* = 0.003
	VOM+	8.15	5.10	
Superficial Surgical Site Infection (%)	VOM−	0.01	0.11	*p* = 0.702
	VOM+	0.00	0.00	
Deep Surgical Site Infection (%)	VOM−	0.01	0.12	*p* = 0.664
	VOM+	0.00	0.00	
Mechanical Complication (%)	VOM−	0.02	0.17	*p* = 0.632
	VOM+	0.00	0.00	

**Table 4:** Baseline radiographic comparison of VOM+ and VOM− patients.

Baseline Radiographic Parameters	Cohort	Mean	Std. Deviation	Significance
Plumbline C7 to S1 (SVA)	VOM−	24.64	50.94	*p* = 0.071
	VOM+	33.34	49.05	
Pelvic Tilt (PT)	VOM−	20.50	10.21	*p* = 0.029
	VOM+	22.74	9.39	
Pelvic Incidence (PI)	VOM−	54.32	12.81	*p* = 0.045
	VOM+	56.82	11.95	
Pelvic Incidence—Lumbar Lordosis (PI-LL)	VOM−	2.81	14.34	*p* = 0.105
	VOM+	4.93	14.75	

**Table 5:** Two-year HRQL comparison of VOM+ and VOM− patients.

2Y Mean HRQL Measures	Cohort	Mean	Std. Deviation	Significance
EQ5D-VAS	VOM−	66.70	19.54	*p* = 0.312
	VOM+	57.60	28.09	
NRS-Back	VOM−	5.53	2.85	*p* = 0.500
	VOM+	6.40	2.70	
NRS-Neck	VOM−	3.78	3.22	*p* = 0.502
	VOM+	2.80	2.68	
NRS-Arm	VOM−	2.40	2.90	*p* = 0.176
	VOM+	4.20	3.70	
NRS-Leg	VOM−	4.53	3.35	*p* = 0.481
	VOM+	4.60	2.88	
Oswestry Disability Index (ODI)	VOM−	45.96	21.43	*p* = 0.726
	VOM+	54.40	31.09	

## Data Availability

The data that support the findings of this study are not publicly, but available from the corresponding author upon reasonable request.
